# Loss of Social Behaviours in Populations of *Pseudomonas aeruginosa* Infecting Lungs of Patients with Cystic Fibrosis

**DOI:** 10.1371/journal.pone.0083124

**Published:** 2014-01-14

**Authors:** Natalie Jiricny, Søren Molin, Kevin Foster, Stephen P. Diggle, Pauline D. Scanlan, Melanie Ghoul, Helle Krogh Johansen, Lorenzo A. Santorelli, Roman Popat, Stuart A. West, Ashleigh S. Griffin

**Affiliations:** 1 Department of Zoology, Oxford University, Oxford, United Kingdom; 2 Department of Systems Biology, Technical University of Denmark, Lyngby, Denmark; 3 School of Life Sciences, Centre for Biomolecular Sciences, University Park, University of Nottingham, Nottingham, United Kingdom; 4 Institute for International Health, Immunology and Microbiology, University of Copenhagen, Copenhagen, Denmark; 5 Centre for Immunity, Infection and Evolution, University of Edinburgh Ashworth Labs, King's Buildings, West Mains Road, Edinburgh, United Kingdom; The Scripps Research Institute and Sorrento Therapeutics, Inc., United States of America

## Abstract

*Pseudomonas aeruginosa*, is an opportunistic, bacterial pathogen causing persistent and frequently fatal infections of the lung in patients with cystic fibrosis. Isolates from chronic infections differ from laboratory and environmental strains in a range of traits and this is widely interpreted as the result of adaptation to the lung environment. Typically, chronic strains carry mutations in global regulation factors that could effect reduced expression of social traits, raising the possibility that competitive dynamics between cooperative and selfish, cheating strains could also drive changes in *P. aeruginosa* infections. We compared the expression of cooperative traits - biofilm formation, secretion of exo-products and quorum sensing (QS) - in *P. aeruginosa* isolates that were estimated to have spent different lengths of time in the lung based on clinical information. All three exo-products involved in nutrient acquisition were produced in significantly smaller quantities with increased duration of infection, and patterns across four QS signal molecules were consistent with accumulation over time of mutations in *lasR*, which are known to disrupt the ability of cells to respond to QS signal. Pyocyanin production, and the proportion of cells in biofilm relative to motile, free-living cells in liquid culture, did not change. Overall, our results confirm that the loss of social behaviour is a consistent trend with time spent in the lung and suggest that social dynamics are potentially relevant to understanding the behaviour of *P. aeruginosa* in lung infections.

## Introduction

Research at the interface of microbiology and evolutionary biology has revealed that populations of bacterial cells are complex communities of individuals that communicate, compete and cooperate with one another [1], [2]. Many basic functions of bacterial cells involve interaction with neighbouring cells through the release of extra-cellular products. Biofilm formation, for example, involves the production of exo-polysaccharides, nutrient acquisition involves the release of degrading enzymes and iron chelators, and cell-to-cell communication involves the release of quorum sensing (QS) signal molecules. Exo-products are often costly to produce, and laboratory experiments show that cells which cheat by avoiding these costs, can outcompete cooperative neighbours, resulting in reduced survival and viability of the population as a whole [3], [4], [5]. The competitive dynamics of cooperators and cheats have been the subject of detailed study in the lab, but little is known about these dynamics in natural populations. In particular, very little is known about social behaviours during pathogenic infections, despite the fact that many virulence factors involve the release of exo-products and are, therefore, potentially subject to exploitation [6], [7].

The bacterium, *Pseudomonas aeruginosa*, has emerged as a model experimental system for studying social behaviour using strains that either cheat or cooperate to perform a number of important functions. *P. aeruginosa* is also widely studied as an opportunistic pathogen of plants, animals and humans and in particular, as a pathogen causing persistent and frequently fatal lung infections in patients with cystic fibrosis (CF) [8]. There is, however, a lack of studies combining these two fields of research to understand how social evolution influences the behaviour of *P. aeruginosa* as a pathogen [6], [9], [10].

There is evidence to suggest that a number of traits that have been shown to be cooperative and exploitable in laboratory cultures, may not be expressed, or have reduced expression in strains isolated from the sputa of CF patients with long-term *P. aeruginosa* infections: biofilm formation [11], the siderophore pyoverdin [12], exo-polysaccharide [13], [14], pyocins [15]; pyocyanin [16], and quorum sensing signal molecules [17], [18], [19]. The evidence is inconsistent, however other studies show that the ability to form biofilms is maintained in chronic isolates [20] and that exo-polysaccharide production is increased in chronic infections [21]. Furthermore, sequence analysis shows that mutations found to accumulate in lung isolates are primarily involved in regulation, making it difficult to assess what phenotypic characteristics are beneficial in the lung environment [18], [22], [23], [24].

The loss of cooperative traits has been shown to confer a competitive advantage over cooperative, wildtype strains in the lab under certain conditions, raising the possibility that the same competitive dynamics are favouring invasion of non-cooperative cheats in the lung [3], [5], [6], [25]. These observations are currently understood, however, to result exclusively from adaptation to the lung environment following colonisation from outside the lung [22], [23], [24], [26]. One difficulty in distinguishing possible explanations arises from the lack of studies that (1) explicitly characterise how putatively social traits change with the duration of time spent in the lung, (2) assess whether there is a general trend across putatively social traits, and (3) determine to what extent this trend is replicated across patients.

In this paper, we report the results of a series of assays designed to provide a general picture of how traits demonstrated to be cooperative in the lab [3], [4], [5], [27] are expressed in strains isolated from different stages of infection. Our sample included isolates from the sputa samples of sixteen patients and eleven isolates from outside the lung (naïve). By treating patients as independent data points, we avoid the common problem of pseudoreplication that results from treating sputa samples or isolates from the same patient as independent data points. As it is not possible without frequent and comprehensive sampling regime to determine the exact length of time a particular genotype has spent in the lung, we estimated this from the clinically defined duration of infection. The strains used in our study were either naïve strains isolated from outside the lung or from three different clinically defined stages of CF lung infection: acute - isolates that have caused a single, acute infection in a child with CF; chronic (<6 months) - isolates that have caused a single, chronic infection for less than six months in an adult with CF; and chronic (>6 months) isolates that have caused chronic infection of more than six month duration in adults with CF (Table S1 in File S1). We assayed three categories of traits shown to be cooperative and exploitable in the lab: (1) the production of extra-cellular products involved in nutrient uptake – protease, elastase and the iron-chelating siderophore, pyoverdin; (2) biofilm formation – a composite trait involving many genes and (3) communication through the production of QS cell-to-cell signalling molecules.

## Materials and Methods

### Origin of isolates

Our collection of *P. aeruginosa* isolates comprised of 11 harvested from outside the lung, either from the environment or different PA01 strains [28] originally isolated from a burn wound [29], [30], six lung isolates from children with CF, four lung isolates from adult CF patients with chronic infections (<6 months duration) and six lung isolates identified as either the transmissible DK-1 or DK-2 strains [22] (Table S1 in File S1) from adult CF patients with chronic infections of >6 months duration (Table S1 in File S1). All clinical isolates were from sputum samples obtained by endolaryngeal suction if the patients are not sputum producers or by sputum cough [31]. Patients were randomly selected from the Copenhagen CF Centre at Rigshospitalet and all have been previously described elsewhere [32], [33], [34].The sample size for all statistical analyses was 27.

To minimise potential for adaptation to the lab environment following isolation from source, all isolates were stored at −80C until required.

### Classification of isolates

We categorised 40 *P. aeruginosa* isolates according to whether they were isolated from the lung and the duration of infection: naïve (external), acute, chronic (<6 months) or chronic (>6 months) for a number of traits (Table S1 in File S1). Where multiple isolates per category were obtained from the same lung, their trait profiles were averaged before analysis to avoid pseudoreplication [35].

### Measuring pyoverdin production

We prepared isolate cultures from freezer stock in 200 µL volumes of King's B medium (20 g Proteose peptone N°3, 10 mL Glycerol, 1.5 g K_2_HPO_4_.3H_2_O, and 1.5 g MgSO_4_.7H_2_O per litre) in a 96-well microtitre plate incubated at 37°C, 200 rpm for 24 h. After 24 h we transferred 1 µL of each culture to a 200 µL volume of iron-limited CAA (5 g Casamino acids, 1.18 g K_2_HPO_4_.3H_2_O, 0.25 g MgSO_4_.7H_2_O, per litre with 100 µg mL^−1^ human apo-transferrin (Sigma) and 20 mM sodium bicarbonate [36], [37] in a 96-well microtitre plate. The apo-transferrin in the CAA binds iron, triggering siderophore production in iron-starved bacteria. We incubated the cultures statically at 37°C for 24 h and then measured their cell density at an absorbance of 600 nm (*A_600_*) [36], [38], and pyoverdin production in relative fluorescence units (RFU), at an excitation wavelength of 400 nm and an emission wavelength of 460 nm [38] using a fluorimeter (SpectraMax M2, Molecular Devices, UK). We replicated each assay seven times per culture, and calculated the mean pyoverdin production per cell by RFU/*A_600_*. We determined siderotypes using multiplex PCR to identify each isolate's ferripyoverdin receptor profile, as described by Bodilis *et al*. [39] (Figure S1 in File S1).

### Measuring pyocyanin production

We assayed for pyocyanin production based on the method described by Essar *et al.* [40]. We cultured isolates from freezer stock in 10 mL King's B (KB) media (KB medium: 20 g Proteose peptone N°3, 10 mL Glycerol, 1.5 g K_2_HPO_4_.3H_2_O, and 1.5 g MgSO_4_.7H_2_O per litre) in glass 25-mL vials and incubated these for 24 h at 37°C, 180 rpm. We then vortexed and dispensed 100 µL of each culture into 1 mL cuvettes containing 900 µL dH_2_O to measured *A*_600_ and then sub-cultured all isolates into 10 mL fresh KB in 100 mL conical flasks to maximise pyocyanin production, with final cell densities standardised to an *A*_600_ value of 1, using 10 mL of sterile KB as the control. Cultures were then incubated at 37°C, 180 rpm for 18 h before measuring *A*_600_ as above; 10 mL of each culture was transferred into 15 mL Falcon tubes and centrifuged at 1,470 *g* for 15 min. We then filter-sterilised (0.2 µm pore size) the supernatant and aliquoted 5 mL into a second set of Falcon tubes to which was added 3 mL chloroform before being vortexed for 5 sec and centrifuged at 1,120 *g* for 10 min. We transferred 2 mL of the bottom extraction layer of chloroform into a new 15 mL Falcon and added 2 mL 0.2 M HCl to each for re-extraction. After vortexing for 5 sec, we centrifuged these extraction layers at 1,120 *g* for 1 min, transferred 1 mL of each top layer to a 1.5 mL Eppendorf before centrifuging at 15,490 *g* for 1 min and removed 900 µL of the top layer of each Eppendorf to a cuvette to read the *A*_520_ of the pyocyanin in acidic solution, using the control as the blank. We then quantified pyocyanin production per culture as µg.mL^−1^ as (*A*_520_/*A*_600_)17.072 [41] then multiplied the result by 0.66, as only ^2^/_3_ of the chloroform extraction layer was sampled [40], [41].

### Measuring total protease activity

We dispensed 250 µL Azocasein solution (2% Azocasein: Sigma, 2 mM CaCl_2_, 40 mM tris-HCl, pH 7.8) into 2 mL Eppendorfs and added 150 µL filter-sterilised supernatant and the control supernatant (as described in the pyocyanin protocol) to each. We vortexed the Azocasein-treated supernatants for 5 sec before incubating them statically at 37°C for 45 min before stopping the reaction by precipitating out the undigested substrate in 1.2 mL trichloroacetic acid (10%) for 15 min and centrifuging at 15,490 *g* for 10 min. We then added 900 µL of each supernatant to 750 µL 1 M NaOH (1 M) in fresh Eppendorfs, vortexed for 5 sec then transferred 100 µL of each acid-treated supernatant into a cuvette containing 900 µL dH_2_O and read *A*_440_ of each using the control as the blank. We calculated the protease activity of each isolates *A*_440_/*A*_600_ of pre-filtered culture [42], [43].

### Measuring elastase activity

Methods for measuring elastase production was based on Ohman et al. (1980) [44]. We dispensed 900 µL Elastin Congo-Red (ECR) buffer (0.0275 g ECR:Sigma, 1 mM CaCl_2_, 100 mM Tris, pH 7.5) into 2 mL Eppendorf tubes and added 100 µL of filter-sterilised supernatant and the control supernatant (as described in the pyocyanin protocol) to each. We then incubated ECR-treated supernatants at 37°C with shaking for 4 h before spinning-down the ECR granules (13,000 rpm for 1 min), transferring 100 µL supernatant off the top of each Eppendorf into 900 µL dH_2_O in a cuvette and reading *A*_495_ of each, using the control as the blank.

### Extraction of quorum sensing molecules

QS molecules extraction methods based on Ortori et al [45], [46]. Isolates were cultured from freezer stock in 10 mL Luria Broth (LB, Sigma) media in glass 25-mL vials and incubated these for 24 h at 37°C, 180rpm, with 10 mL of sterile LB as the control. Cultures were then vortexed and 100 µL dispensed into 1 mL cuvettes containing 900 µL dH_2_O for measuring cell density at *A*_600_. We then transferred the 9.9 mL of each culture and the control into 15 mL Falcon tubes and centrifuged these at 1,470 *g* for 15 min before filter-sterilising (0.2 µm pore size) the supernatants and aliquoting 9 mL of each into a second set of Falcon tubes. We extracted QS molecules from the supernatants by adding 9.5 mL of acidified ethyl acetate (100 µl glacial acetic acid, 1 L ethyl acetate), vortexing these mixtures for 30 s and then centrifuging them for 10 min at 1,470 *g* to separate the organic and aqueous phases. We then removed 7 mL of the organic phase of each treated supernatant and dried them using a centrifugal evaporator (Jouan, RC10 22) for 2 h at 50°C. We reconstituted the dried samples in 1 mL methanol and aliquoted 500 µl of each into two 1.5 mL Eppendorfs. Samples were stored at −20°C.

### Quantifying proportion of the cell population in biofilm

We quantified the proportion of cells growing as a biofilm in a microtitre plate using a BacTiter-Glo assay (BacTiter-Glo™ Microbial Cell Viability Assay kit, Promega). Cultures were inoculated from freezer stock and grown overnight, in 3 mL Luria Broth (LB) at 37°C, 200 rpm. We normalised the cultures to 0.05 *A*_600_ in 3 mL fresh LB, incubated them for 2 h to reach exponential phase, then diluted them to 0.0025 *A*_600_ in 10 mL 1% Bacto Tryptone media (TB: 10 g Bacto™ Tryptone (Becton, Dickinson & Co, Franklin Lakes, NJ, USA) in 1 L distilled H_2_O (dH_2_O)). We aliquoted eight 150 µL volumes of each culture into a flat-bottomed 96-well microtitre plate (plate A), filling one column with sterile TB as a negative control. Plates were sealed in an airtight container and incubated at room temperature (RT) for 24 h. BacTiter-Glo (BTG) substrate and buffer was thawed and mixed before being left for a further 1 h 30 min to allow time for trace amounts of ATP in the reagent to burn-off – maximising the assay's sensitivity. After 24 h incubation we removed 40 µL of supernatant from each well of plate A and diluted these 1∶2.5 in a 96-well plate containing 60 µL dH_2_O (plate B). We then transferred 66 µL of the plate B dilutions to a black 96-well microtitre plate (plate C), and added 33 µL of BTG. We then washed plate A three times in distilled water (dH_2_O) to remove the remaining supernatants, dried the plate on paper towels and loaded each well with 50 µL equilibrated BTG, quickly followed by 100 µL Phosphate Buffered Saline (PBS: dH_2_O). We then placed plate A on a 750 rpm shaking platform for 5 min and then transferred 100 µL of each well of plate A to a black 96-well plate (plate D). We then read the luminescence per well of plates D and E immediately (*t*_1_) in counts per minute (CPM) on a TopCount.NXT™ Microplate Scintillation & Luminescence counter. A second CPM reading was taken of both plates 2–4 h later (*t*_2_). The two CPM readings were used to calculate the total luminescence emitted by the supernatant and biofilm populations of each culture, and these totals were used to back-calculate signal strength of each population at time 0 when the BTG was first added to each well ([CPM]_0_). [CPM]_0_ was calculated as:
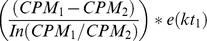
where *k* = ln(CPM_1_/CPM_2_)/(*t_2_*-*t_1_*).

We then corrected the [CPM]_0_ values for dilution factors and calculated the proportion of cells in a microtitre culture existing in biofilm as: biofilm [CPM]_0_/(biofilm [CPM]_0_ + planktonic[CPM]_0_), using the average for the 8 replicates of each isolate for further analysis.

### Quantification of QS signal molecules by liquid chromatography/mass spectrometry (LC/MS)

We prepared reconstituted organic extracts for isolates as described above. After sonicating for 5 min followed by centrifuging at 9,500 *g* for 10 min, we transferred extracted supernatant (described above) of each into a glass insert within a high performance liquid chromatography (HPLC) vial and sealed these with screw caps. For the liquid chromatography (LC) analysis of QS molecules *N-*(3-oxo-dodecanoyl)-L-homoserine lactone (3-oxo-C_12_-HSL), *N-*butanoyl-L-homoserine lactone (C_4_-HSL), 2-heptyl-4(1*H*)-quinolone (C_7_-HHQ), and 2-heptyl-3-hydroxy-4(1*H*)-quinolone (PQS), we used a mobile phase run through a pre-primed and equilibrated HPLC Agilent instrument, series 1200, with an Ascentis Express C18 column (150×2.1 mm internal diameter, 2.7 µm particle size). We then cleaned the Agilent HPLC system and recalibrated it into the positive ion mode to run full mass spectrometry (MS) scans on the samples for the same QS molecule range. We then used the Bruker DataAnalysis programme version 3.3 to interpret the LC/MS data. The programme matched LC retention times and MS peak spectra to the 10 µM QS standards injected at intervals throughout both assay methods to produce an LC/MS profile for each isolate.

### Statistical analyses

**Figure 1:**
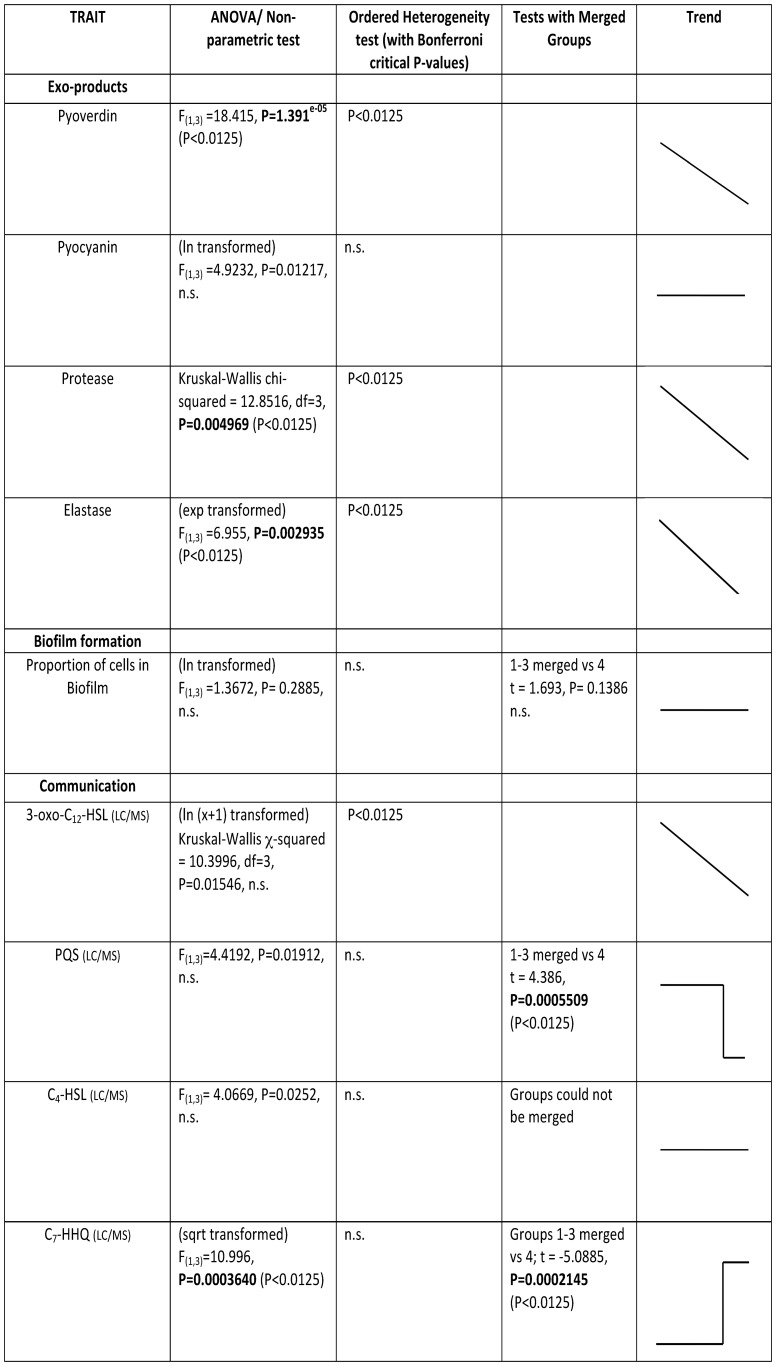
A table summarising statistical analyses and trends in expression over time across all traits.

We tested for significant differences with time spent in the lung (as estimated by source of isolate) using a one-way ANOVA analysis. We normalised residuals prior to analyses: data from biofilm and pyocyanin assys were transformed by natural log (ln(*x*)), and data from the elastase and the C_7_-HHQ assays by exponential (exp(*x*)) and square root (sqrt(*x*)) respectively. We performed Kruskal-Wallis tests for non-parametric data sets to test for significant differences in production of protease and 3-oxo-C_12_-HSL. We log-transformed 3-oxo-C_12_-HSL data by (ln(*x+*1)) to normalise residuals. Ordered heterogeneity (OH) tests were used to test for the occurrence of a trend in the production levels of each trait across the four isolate categories. We merged data that showed no significant differences in production across categories, naïve to chronic (confirmed by Tukey's HSD (honestly significant difference) test); Figure 1) and repeated the above analysis on merged data sets. When the merged data resulted in only two categories, we performed a t-test to check for significant differences, and no OH tests would be required in such cases. To correct for multiple comparisons being applied to the same dataset, the Bonferroni correction was applied to all analyses, with each test on exo-product traits requiring P-values of *P*<0.0125 (0.05/4 tests) and each test of signal molecule production requiring P<0.0125 (0.05/4 tests).

Binomial GLMs were used to check whether there is a significant representation of siderotypes I, II or III in each isolate category. All analysis was implemented in R statistical software (http://www.R-project.org) (Summary of all tests and significance levels reported in Figure 1).

## Results

### Secretion of exo-products

**Figure 2:**
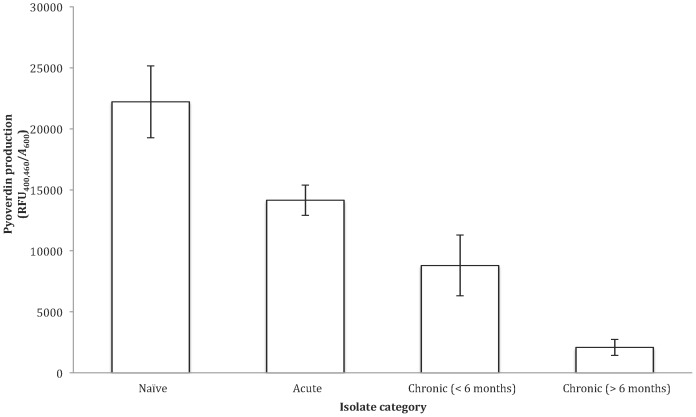
The estimated pyoverdin production per cell of the four *P. aeruginosa* isolate categories. Pyoverdin production calculated as RFU_400,460_/*A*_600_. Data shown as untransformed averages of five replicates per isolate per category ±SE.

**Figure 3:**
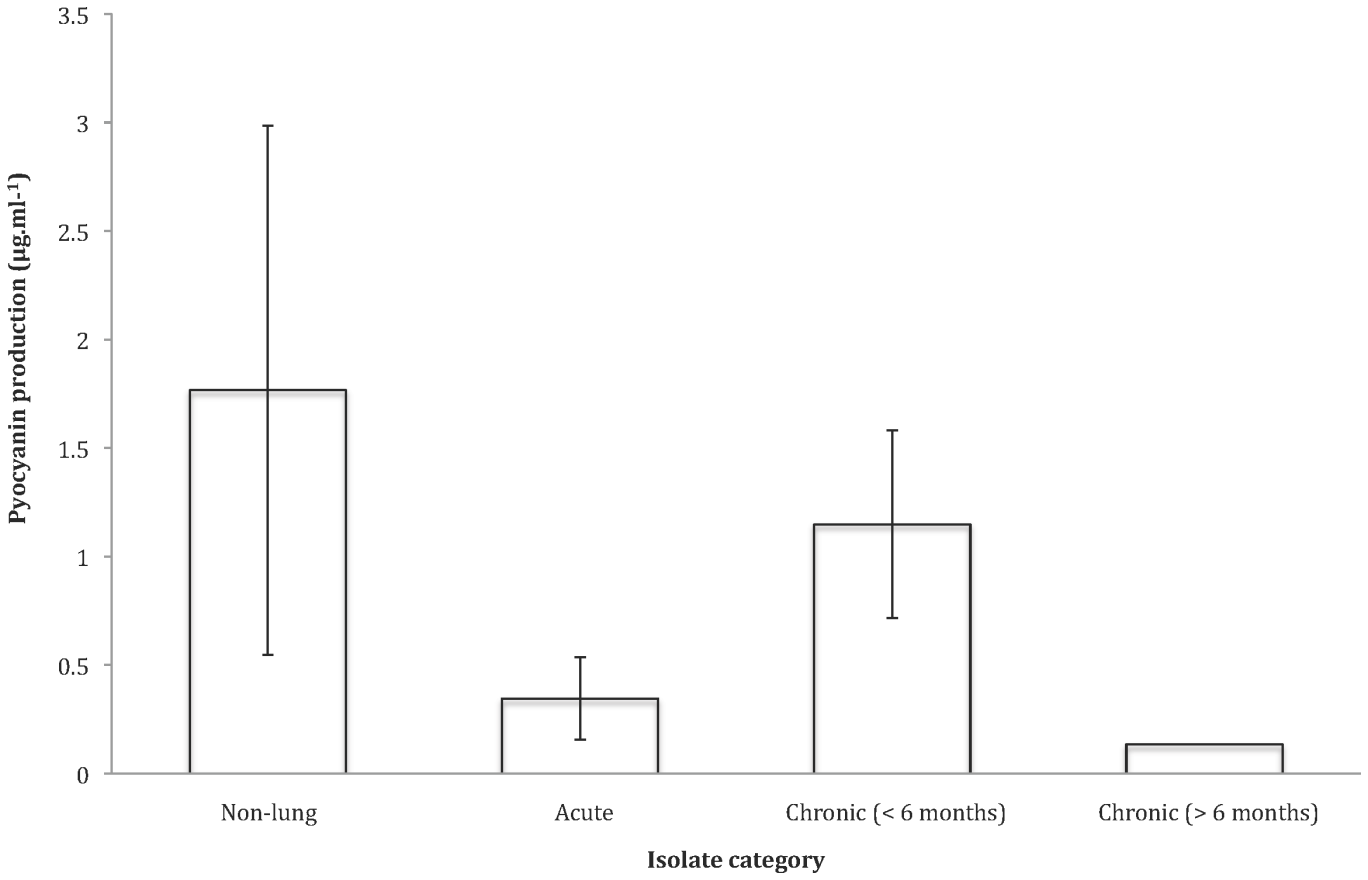
Pyocyanin production of the four *P. aeruginosa* isolate categories. Data shown as untransformed averages of five replicates per isolate per category ±SE.

**Figure 4:**
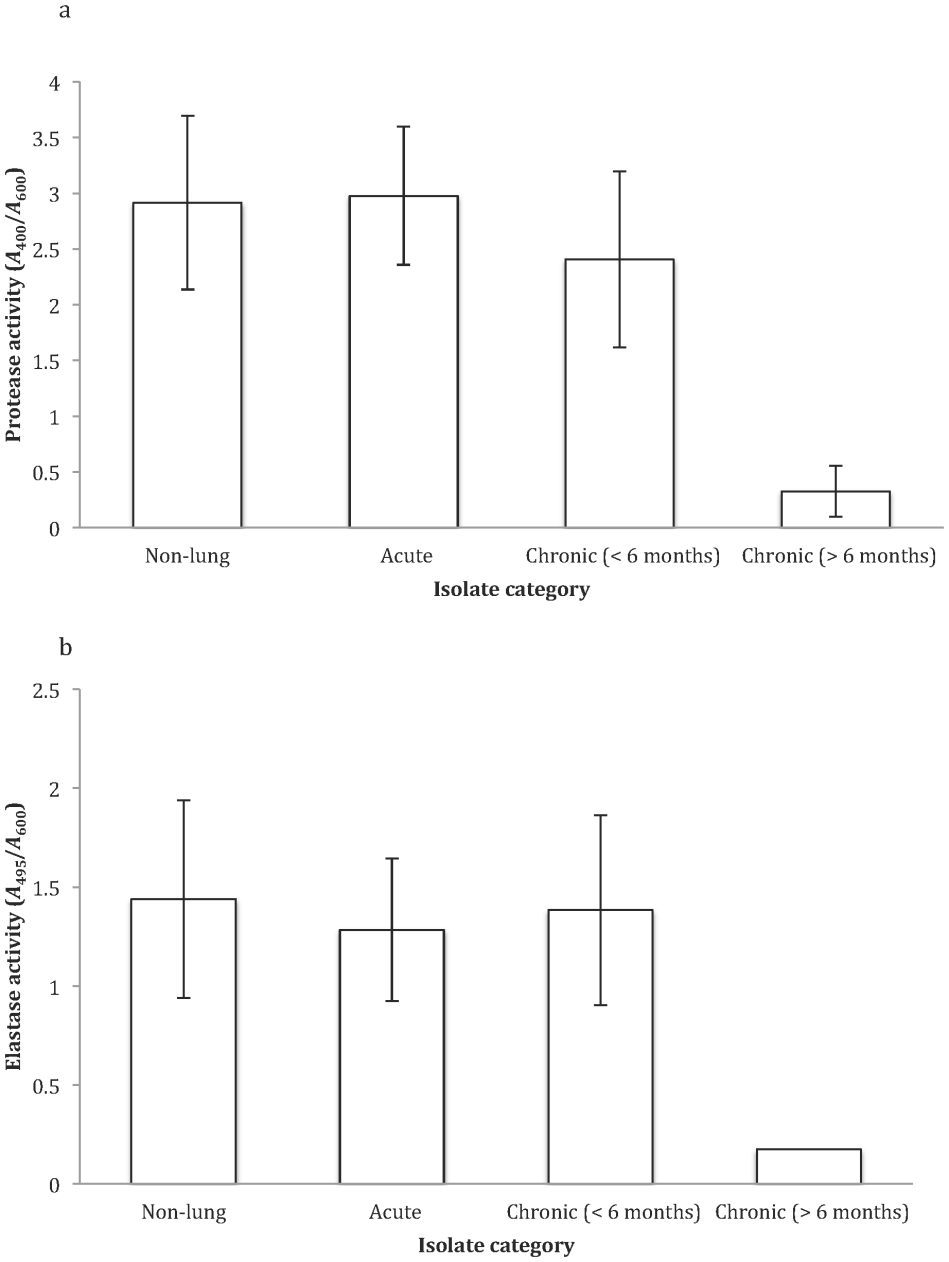
The production of ezymes protease (a) and elastase (b) of the four *P. aeruginosa* isolate categories. Protease and elastase activity calculated as *A*_440_/*A*_600_ and *A*_495_/*A*_600_, respectively. Data shown as untransformed averages of five replicates per isolate per category ±SE.

There was a significant negative correlation between the production of pyoverdin, and stage of infection, with the least productive strains isolated from patients with chronic infections (>6 months) (OH test: *F*_(1, 3)_ = 18.415, *P = *P = 0.000014< Bonferroni (0.0125) Figure 1, Figure 2). Pyocyanin production was not significantly correlated with duration of infection (Figure 1, Figure 3). OH test (ln transformed): *F*_(1, 3)_ = 4.92, *P* = 0.01 > Bonferroni (0.0125)). Both protease and elastase production were significantly reduced with increasing duration of infection (Figure 1, Figures 4a and 4b): protease – Kruskall Wallis χ^2^ = 12.85, p = 0.005<Bonferroni (0.0125); elastase - OH test (exp transformed), *F*_(1, 3)_ = 6.96, p = 0.003 , Bonferroni (0.0125).

### Biofilm Formation

**Figure 5:**
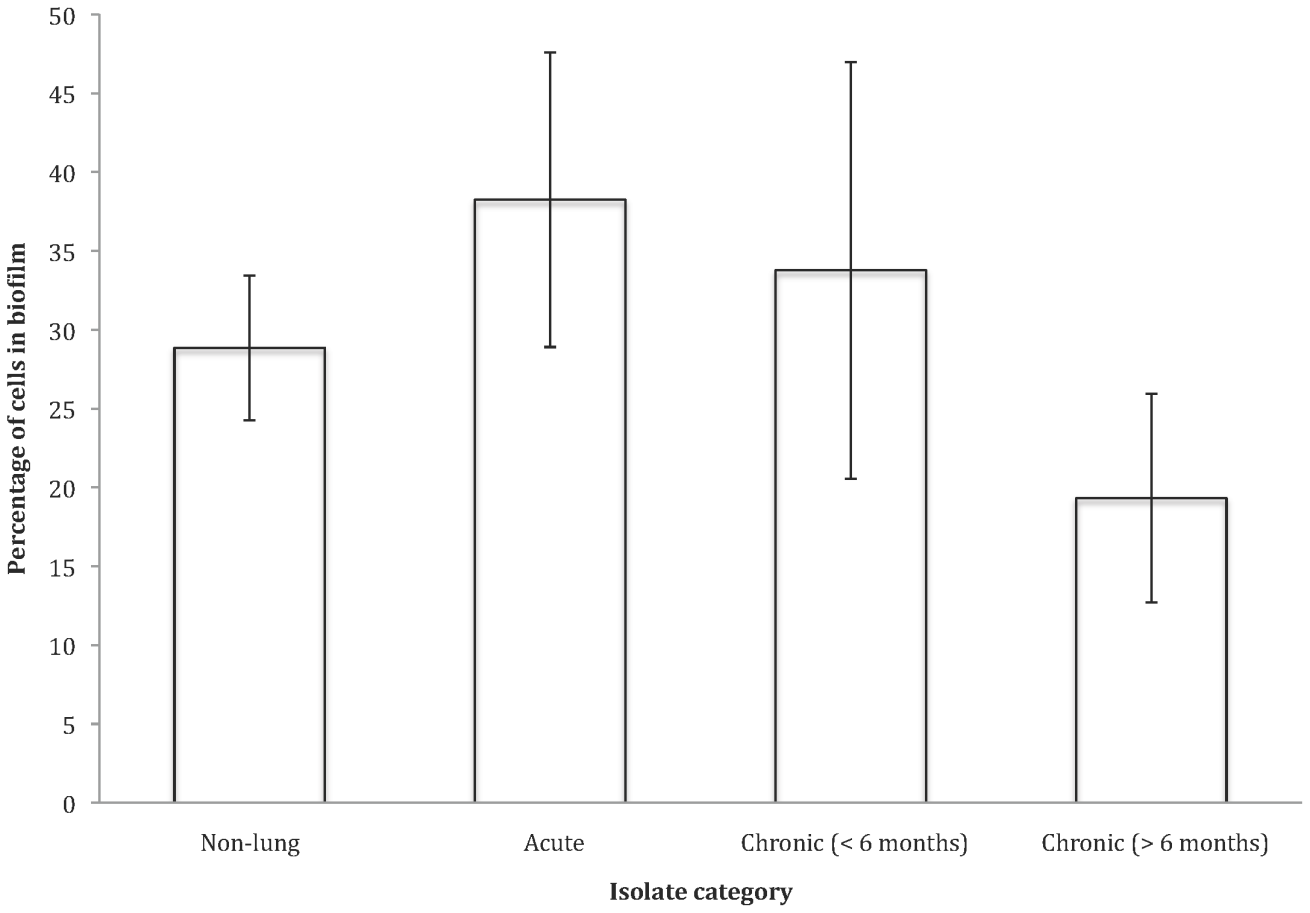
The percentage of cells in microtitre culture existing in biofilm of the four *P. aeruginosa* isolate categories. Data shown as untransformed averages of five replicates per isolate per category ±SE.

There was no significant difference in the proportion of cells in biofilm, relative to growing in suspension, across categories (OH test (ln transformed): *F*_(1, 3)_ = 1.37, *P* = 0.29 > Bonferroni (0.05) Figure 1, Figure 5), suggesting that the propensity of cells to form biofilm is not reduced with duration of infection.

### Communication

**Figure 6:**
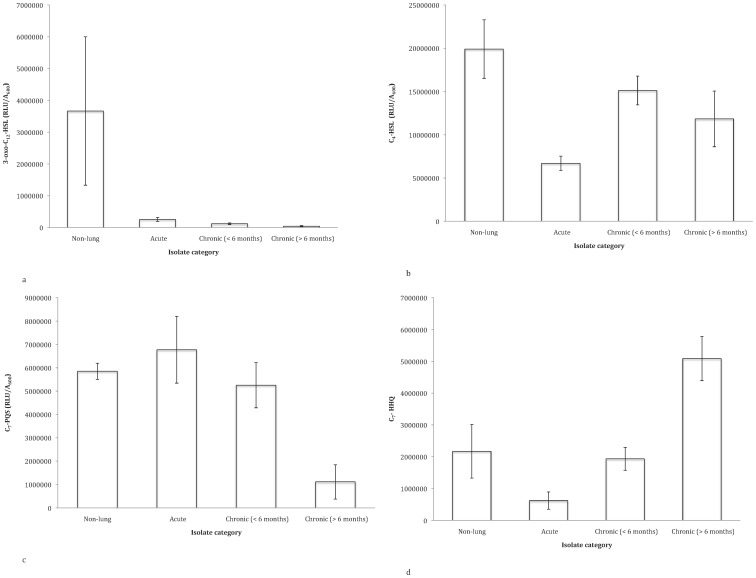
Relative production of (a) 3-oxo-C12-HSL and (b) C_4_-HSL (c) PQS and (d) C_7_-HHQ interpreted from LC/MS peak readings. Data shown as untransformed averages of single replicates per isolate per category ±SE.

We found evidence for reduced production of two out of four quorum sensing signal molecules that we assayed with increased duration of infection (Figure 1, Figure 6a–6d): 3-oxo-C_12_-HSL showed a significantly negative trend with increased duration of infection when assayed using LC/MS (ordered heterogeneity test P<,0.001 Bonferroni (0.0125)). We also found that PQS was significantly reduced in strains isolated from chronic infections (>6 months) relative to isolates in the other three categories (t = 4.386, p = 0.0006, Bonferroni (0.0125). In contrast, C_4_-HSL showed no trend and the production of C_7_-HHQ in strains isolated from patients with chronic infections (>6 months) was significantly greater than isolates from other categories (t = 2.27, P = 0.0002, Bonferroni (0.0125); Figure 1).

## Discussion

We examined a range of social traits in *P. aeruginosa* strains isolated from outside and inside the lungs of cystic fibrosis patients that were estimated to have been infected for different lengths of time. We found that: (1) pyoverdin, total protease and elastase production were reduced with increased duration of infection (Figures 1, 2, and 3); (2) the ability of cells to form biofilm showed no significant change with increased duration of infection (Figure 5); and (3) while the production of the QS signals 3-oxo-C12-HSL and PQS declined with increased duration of infection (Figures 6a and 6c), the production of C4-HSL did not change and HHQ increased (Figure 6b and 6d). Overall, these results suggest that a number of *P. aeruginosa* social traits, but not all, are lost over time, during the lung infections of cystic fibrosis patients. These changes could occur as a result of evolution within a lineage, or by displacement of one by another lineage – for example, chronic infections of >6 months are typically caused by one of two strains known to be transmissible between patients in the Danish CF community [22] (Table S1 in File S1).

The extent to which cooperative traits are vulnerable to exploitation is expected to depend on several factors and these factors are likely to vary widely between traits. For example, the ability of one cell to exploit a public good produced by another cell will depend on the diffusion distance of the molecule, the spatial structuring of the environment, and the ability of cells to produce exo-products which can only be utilised by cells of the same genotype. Specificity is likely to vary between traits: it has been shown that members of different species benefit from information obtained from signalling systems, the production of beta-lactamases. On the other hand, it has been shown that strains of *P. aeruginosa* produce pyoverdin molecules which produce iron-complexes that can only be taken up by cells of the same strain. High levels of diversifying selection at *pvd* syntheses loci are consistent with the possibility that this is a mechanism to prevent exploitation. Evidently, therefore, it is extremely challenging to make predictions about whether a population of cooperative bacteria is vulnerable to invasion by cheats. We would argue, however, that the possibility that interspecific competitive dynamics are at least partially responsible for the phenotypic patterns described in this paper is important to consider. No natural population of organisms has yet been discovered to have evolved entirely in response to its environment, without the additional influence of intra- and inter-specific interactions.

In interpreting our observations, it is important to consider that the traits we measured are unlikely to vary independently from one another. Many of the trends we observed suggest the accumulation of *lasR* QS mutants with increasing duration of infection, a finding in common with previous studies [17], [18], [24]. We found a decrease in total protease, elastase and the autoinducer 3-oxo-C_12_-HSL, the production of which are absent or greatly reduced in *lasR* mutants [47], [48]. We also noted a decrease in PQS and an increase in HHQ. Again an accumulation of *lasR* mutants could explain these results. The production of HHQ is regulated by the *pqsABCDE* operon and conversion of HHQ into PQS is via PqsH, which is under the control of LasR [49]. We did not observe a trend in production of either the *rhl* QS signal molecule C_4_-HSL or the *rhl-*dependent phenazine pyocyanin. It is known that activity of the *rhl-*system is only delayed in a *lasR* mutant and both C4-HSL and pyocyanin have been shown to be produced in the absence of LasR [50], [51]. Complex interactions between loci emphasises the importance of studying phenotypic as well as genetic patterns as the presence or absence of a gene may not necessarily effect phenotype and therefore exposure to selection [52].

A remaining challenge is to characterise the selective environment experienced by bacterial cells living in the lung environment. It is likely that the phenotypes of bacterial cells living inside the lung are largely determined by selection for antibiotic resistance, but the lung is a complex environment with multiple niches, which change during the course of an infection [22]. It is clear, however, that not all of the patterns we observed in this study are easily explained by adaptation alone. For example, production of the iron-chelator, pyoverdin, is predicted to decrease with availability of free iron in the environment, but we find that pyoverdin production is relatively high in isolates from acute infections where iron levels have been shown to be highest [53]. Similarly, production of the QS signal molecule PQS is specifically involved in evasion of the immune system by triggering defence mechanisms against polymorphonuclear phagocytes, and suppressing the activation of T-cells [54] but we find that PQS is reduced with duration of infection. Competition between cooperative and cheating strains, selecting for mutations that reduce investment in cooperative behaviours, could provide an additional explanation for observed changes in behaviour.

A primary aim of research into the behaviour of *P. aeruginosa* in the CF lung is to improve treatment strategies. Potentially the most important difference between a community shaped by adaptation to the lung and a community shaped by social interactions is the effect on transmission between patients. Cheats are expected to be poor at establishing a novel infection but have resistance to invasion from wild-type cooperative strains that will quickly be exploited. The highly transmissible Liverpool Epidemic Strains LES, has been characterised as having highly up-regulated quorum sensing systems but show similar patterns of down-regulation in strains isolated from chronic infections [55]. This suggests that cheating could reduce transmission but also raises questions about where highly transmissible strains originate from and how they are able to persist in populations dominated by QS mutants that have been shown to be competitively superior in the lab [3]. Taking social interactions into account could not only help explain the phenotypes observed in chronic infections but also why infections seem to be initiated by highly cooperative strains.

## Supporting Information

File S1 **Supporting information includes list of isolates in Table S1 and section on siderotype analysis.** Data from siderotype analysis presented in Figure S1. Section 1: Categorisation of *P. aeruginosa* isolates; Table S1. Section 2: Results of siderotype analysis; Figure S1.
(DOCX)
