# Supplementary material for: Loss of Social Behaviours in Populations of Pseudomonas aeruginosa Infecting Lungs of Patients with Cystic Fibrosis
Source: PLoS One. 2014 Jan 14;9(1):e83124. doi: 10.1371/journal.pone.0083124 (PMC3891558; doi:10.1371/journal.pone.0083124)
Supplement: File S1 — Supporting information includes list of isolates in Table S1 and section on siderotype analysis. Data from siderotype analysis presented in Figure S1. Section 1: Categorisation of P. aeruginosa isolates; Table S1. Section 2: Results of siderotype analysis; Figure S1. (DOCX) [file pone.0083124.s001.docx]

**Supplementary Information**

Jiricny, N *et al.* Loss of social behaviours in populations of *Pseudomonas aeruginosa* infecting lungs of patients with cystic fibrosis

**Contents**

**Section 1: Categorisation of *P. aeruginosa* isolates; Table S1**

**Section 2: Results of siderotype analysis; Figure S1**

**Section 1: Characterisation of the *P. aeruginosa* isolates; Table S1**

| **Isolate category** | **Isolate name** | **Isolate source** |
| --- | --- | --- |
| Naïve | PAO6049 | Lab PAO1 |
|  | ATCC15692 | Lab PAO1 |
|  | P3 | Lab PAO1 |
|  | P76 | Lab PAO1 |
|  | P100 | Lab PAO1 |
|  | P93 | Lab PAO1 |
|  | Mt St Helen's | Environment |
|  | R4 | Sink |
|  | R5 | Sink |
|  | F-2* | Water isolate |
|  | F-3* | Water isolate |
| Acute infection | B3-0 | Child with CF #3 |
|  | B6-0^*^ | Child with CF #6 |
|  | B6-2 | Child with CF #6 |
|  | B6-3 | Child with CF #6 |
|  | B7-1 | Child with CF #7 |
|  | B7-3 | Child with CF#7 |
|  | B11-1^**^ | Child with CF #11 |
|  | B12-0^*^ | Child with CF #12 |
|  | B13-2^**^ | Child with CF #13 |
|  | B28-1^**^ | Child with CF #28 |
| Chronic infection (< 6 months) | 2-1 | Adult with CF #2 |
|  | 2-3 | Adult with CF #2 |
|  | 6-2 | Adult with CF #6 |
|  | 11-1 | Adult with CF #11 |
|  | 11-6 | Adult with CF #11 |
|  | 11-7 | Adult with CF #11 |
|  | 16-1 | Adult with CF #16 |
|  | 16-2 | Adult with CF #16 |
|  | 16-10 | Adult with CF #16 |
|  | 16-14 | Adult with CF #16 |
| Chronic infection (> 6 months) | DK-1^Ɨ^ | Adult with CF #2 |
|  | DK-2 ^Ɨ^ | Adult with CF #2 |
|  | DK-2 | Adult with CF #6 |
|  | DK-1 | Adult with CF #6 |
|  | DK-2 | Adult with CF #7 |
|  | DK-2 | Adult with CF #10 |
|  | DK-1 | Adult with CF #10 |
|  | DK-2 | Adult with CF #11 |
|  | DK-2 | Adult with CF #16 |

* These isolates were not included in the sample assayed for QS signal molecule production or proportion of cells in biofilm as a result of date of acquisition.

^Ɨ^ Bjarnsholt, T., Jensen, P.O., Jakobsen, T.H., Phipps, R., Nielsen, A.K., Rybtke, M.T., Tolker-Nielsen, T., Givskov, M., Hoiby, N. & Ciofu, O. 2010. Quorum sensing and virulence of *Pseudomonas aeruginosa* during lung infection of cystic fibrosis patients. *PLoS ONE* **5:** e10115.doi:10110.11371/journal.pone.0010115.

^*^ Rau, M.H., Hansen, S.K., Johansen, H.K., Thomsen, L.E., Workman, C.T., Nielsen, K.F., Jelsbak, L., Hoiby, N., Yang, L. & Molin, S. 2010. Early adaptive developments of *Pseudomnas aeruginosa* after the transition from life in the environment to persistent colonization in the airways of cystic fibrosis hosts. *Environmental Microbiology* **12:** 1643-1658.

^**^ Krogh Johansen, H (unpublished). All other clinical isolates: Jelsbak, L, Krogh Johansen, H, Forst, A, Thøgersen, R, Thomsen, L.E., Ciou, O, Yang, L, Haagensen, JAJ, Høiby, N, Molin, S (2007) Molecular epidemiology and dynamics of Pseudomonas aeruginosa populations in lungs of cystic fibrosis patients. Infection and Immunity 75, 2214-2224.

**Section 2: Siderotype Analysis**

We determined siderotypes using multiplex PCR to identify each isolate’s ferripyoverdin receptor profile, as described by Bodilis *et al*. (23).

Figure S1: Siderotype representation within the four *P. aeruginosa* isolate categories. Siderotypes are: I (black), II (grey) and III (white).

There was a significant decrease in the proportion of strains with siderotype I over time in the lung (Binomial GLM, P=0.00086, d.f.= 20) and a significant increase in the proportion of strains with siderotype II (Binomial GLM, P= 0.000945 df = 20). In the multiple chronic infection category of strains only siderotype II was found (Figure S1), and siderotype II was only found in samples taken from the lung. The ferripyoverdin profiles of isolates B6-2, B12-0, 2-1 and 16-1 (Table S1) were ambiguous so we could not include them in statistical analyses.
